# M.I.C.E—Mental Health Intervention for Children with Epilepsy: a randomised controlled, multi-centre clinical trial evaluating the clinical and cost-effectiveness of MATCH-ADTC in addition to usual care compared to usual care alone for children and young people with common mental health disorders and epilepsy—study protocol

**DOI:** 10.1186/s13063-020-05003-9

**Published:** 2021-02-11

**Authors:** Sophie D. Bennett, J. Helen Cross, Anna E. Coughtrey, Isobel Heyman, Tamsin Ford, Bruce Chorpita, Rona Moss-Morris, Sarah Byford, Emma Dalrymple, Colin Reilly, Terence Stephenson, Caroline Doré, Sophia Varadkar, James Blackstone, Kashfia Chowdhury, Poushali Ganguli, Liz Deane, MICE Study Team, Peter Fonagy, Jonathan Smith, Roz Shafran

**Affiliations:** 1 UCL Great Ormond Street Institute of Child Health, 30 Guilford Street, London, WC1N 1EH UK; 2 Great Ormond Street Hospital NHS Foundation Trust, London, UK; 3 UCL Great Ormond Street Institute of Child Health, London, UK; 4 Department of Psychiatry, Cambridge University, Cambridge, UK; 5 Cambridge and Peterborough NHS Foundation Trust, Cambridge, UK; 6 UCLA , California Los Angeles, USA; 7 Institute of Psychiatry, Psychology & Neuroscience, King’s College London, London, UK; 8 National Centre for Young People with Epilepsy, Surrey, UK; 9 Comprehensive Clinical Trials Unit, University College London, London, UK

**Keywords:** Epilepsy, Neurology, Paediatric, Mental health, Anxiety, Depression, Disruptive behaviour, Cognitive behaviour therapy, Teletherapy

## Abstract

**Background:**

Mental health disorders in the context of long-term conditions in children and young people are currently overlooked and undertreated. Evidence-based psychological treatments for common childhood mental health disorders (anxiety, depression and disruptive behaviour disorders) have not been systematically evaluated in young people with epilepsy despite their high prevalence in this population. The aim of this multi-site randomised controlled trial is to determine the clinical and cost-effectiveness of adding a modular psychological intervention to usual care for the mental health disorders in comparison to assessment-enhanced usual care alone.

**Methods:**

In total, 334 participants aged 3–18 years attending epilepsy services will be screened for mental health disorders with the Strengths and Difficulties Questionnaire (SDQ) and the diagnostic Development and Wellbeing Assessment (DAWBA). Those identified as having a mental health disorder and consenting to the trial will be randomised to either receive up to 22 sessions of the modular psychological intervention (MATCH-ADTC) delivered over the telephone over 6 months by non-mental health professionals in addition to usual care or to assessment-enhanced usual care alone. Outcomes will be measured at baseline, 6 months and 12 months post-randomisation. It is hypothesised that MATCH-ADTC plus usual care will be superior to assessment-enhanced usual care in improving emotional and behavioural symptoms. The primary outcome is the SDQ reported by parents at 6 months. Secondary outcomes include parent-reported mental health measures such as the Revised Children’s Anxiety and Depression Scale, quality of life measures such as the Paediatric Quality of Life Inventory and physical health measures such as the Hague Seizure Severity Scale. Outcome assessors will be blinded to group assignment. Qualitative process evaluations and a health economic evaluation will also be completed.

**Discussion:**

This trial aims to determine whether a systematic and integrated approach to the identification and treatment of mental health disorders in children and young people with epilepsy is clinically and cost-effective. The findings will contribute to policies and practice with regard to addressing mental health needs in children and young people with other long-term conditions.

**Trial registration:**

ISRCTN ISRCTN57823197. Registered on 25 February 2019.

## Administrative information

**Table:** 

Title {1}	M.I.C.E—Mental Health Intervention for Children with Epilepsy A randomised controlled, multi-centre clinical trial evaluating the clinical and cost-effectiveness of MATCH-ADTC in addition to usual care compared to usual care alone for children and young people with common mental health disorders and epilepsy
Trial registration {2a and 2b}.	Primary Registry and Trial Identifying Number ISRCTN57823197; Date of registration in primary registry 25th February 2019
Protocol version {3}	V4.0 27-July-2020
Funding {4}	MICE is fully funded by an NIHR Programme Grant for Applied Research RP-PG-0616-20007. It is not expected that any further external funding will be sought.
Author details {5a}	Sophie D. Bennett, J Helen Cross, Anna E. Coughtrey, Isobel Heyman, Tamsin Ford, Rona Moss-Morris, Sarah Byford, Emma Dalrymple, Colin Reilly, Terence Stephenson, Caroline Doré, Sophia Varadkar, James Blackstone, Kashfia Chowdhury, Poushali Ganguli, Liz Deane, MICE Study Team and Roz Shafran.
Name and contact information for the trial sponsor {5b}	Great Ormond Street Hospital for Children NHS Foundation Trust Joint R&D Office GOSH/ICH, based at UCL Great Ormond Street Institute of Child Health, 30 Guilford Street, London, WC1N 1EH, United Kingdom
Role of sponsor {5c}	The role of the sponsor is to take on responsibility for securing the arrangements to initiate, manage and finance the trial.

## Background and rationale {6a}

Children and young people with epilepsy who also have mental ill-health are poorly served by current health care provision. The mental health disorders often go undetected and undiagnosed, treatment when provided is often inadequate and service organisation fails to integrate and co-locate physical and mental health care [1]. This is despite the evidence that co-occurring mental ill-health impacts on physical health and quality of life and, in adults, increases chance of death [2, 3].

The protocol described here is for a randomised controlled trial (RCT) designed to address aspects of this problem. The clinical trial forms Work Package 3 of a broader research programme aimed at developing a new psychological treatment for anxiety, depression and behavioural problems in children with epilepsy.

The background and rationale for the research programme as a whole is embedded within a wider national priority within the UK National Health Service (NHS) - namely the closer integration of mental and physical health care [4]. Epilepsy is the most common serious neurological disorder in childhood, affecting approximately 1 in 150 children [5]. Evidence-based psychological treatments for common childhood mental health disorders (anxiety, depression and disruptive behaviour disorders) have not been systematically evaluated in young people with epilepsy despite their high prevalence in this population [6–9]. Although epilepsy is defined by recurrent seizures, the comorbid mental health disorders such as anxiety and depression often have a greater impact on quality of life [10]. One reason for the failure to apply psychological treatments may be current service organisation. Such patients are seen in paediatric services and, if mental health input is needed, are referred to local Child and Adolescent Mental Health Services (CAMHS). However, CAMHS are overstretched [11] and inexperienced in dealing with mental ill-health in the context of a long-term condition [12].

The Modular Approach to Therapy for Children with Anxiety, Depression, Trauma or Conduct problems (MATCH-ADTC) is a psychological intervention which is compliant with UK recommended best practice with modules that are selected to treat multiple common mental health disorders in youth [13–15]. It draws from psychological theories to encourage positive behaviours and reduce unhelpful ones and is based on principles of cognitive behaviour therapy (CBT) for managing behaviour problems, anxiety, depression and trauma in children. The treatment contains therapeutic procedures that were selected to correspond to the practices found in several leading evidence-based psychological treatments (EBTs), against which MATCH was compared in randomised trials [14, 15]. Despite extensive trial literature demonstrating the efficacy of evidence-based psychological treatments [16], they are disappointingly rarely implemented in clinical practice [17]. The MATCH-ADTC protocol and manual modular design ensures that the needs of individuals with multiple mental disorders (40% of patients) are met - in contrast to the protocol for the majority of trials which address only single, specific disorders [18].

MATCH-ADTC is divided into 33 practice modules that correspond to four focus areas with corresponding coordinating modules. The first focus area/coordinating module is for anxiety disorders and the practice modules within this correspond to the strategies in ‘Coping Cat’ [19]. The second focus area is for the treatment of depression and the modules within this corresponds to the strategies in ‘Primary and Secondary Control Enhancement Training’ (PASCET) [20]. The third focus area is for Parent Training for behavioural problems [21]. The fourth focus area is for trauma. Both Coping Cat and PASCET have been used successfully in children with physical illnesses and comorbid mental health conditions [22]. According to the protocol, the therapist focuses on the initial problem area identified by the patient and family together with the information gathered in the clinical and research assessment. It is considered to represent true evidence-based practice in that the protocol combines research evidence, clinical judgement and patient values and preferences [23]. Once a problem area has been selected (e.g. anxiety), an algorithm specifies a default sequence of focus area and practice modules and guides clinical judgement. However, the intervention is also personalised so that if the default sequence cannot be implemented (e.g. due to low mood), then the sequence can be changed to address the immediate issue. Once the interfering issue has been addressed, treatment for the original problem area is resumed. Carers and family members can join the telephone/video conferencing sessions where appropriate and in the original trial 41% of the sessions included the child plus a family member [14].

Focus groups with patients and family members identified the need for an epilepsy-specific focus area/coordinating module, which was not a component of the published MATCH-ADTC protocol. This was felt necessary to meet the needs of young people with both epilepsy and mental health disorders. Epilepsy-relevant content was integrated throughout the treatment, such as epilepsy-related examples in worksheets. The epilepsy materials were developed and finalised in earlier stages of the programme of research [24].

### Objectives {7}

The overarching aim of this programme of research is to transform the treatment of mental health disorders in young people with epilepsy by systematically identifying and treating mental health problems from within epilepsy services to enable early detection and intervention.

Specifically, the trial aims to determine the clinical and cost-effectiveness of adding a personalised modular psychological intervention—MATCH-ADTC with epilepsy-relevant content integrated throughout and an additional epilepsy-specific focus area, with one compulsory and three optional modules—to assessment-enhanced usual care. Assessment-enhanced usual care is usual care with the addition of standardised assessment measures, since those in this arm will have completed the Development and Well-being Assessment (DAWBA) and the results of the DAWBA will be provided to clinicians and to participants. The intervention will be delivered by non-mental health specialists, over the telephone/online, within epilepsy services, for young people with epilepsy who also meet Diagnostic and Statistical Manual of Mental Disorders (DSM-5) diagnostic criteria for a mental health disorder.

### Trial design {8}

This study is a two-arm randomised controlled superiority trial comparing the clinical and cost-effectiveness of MATCH-ADTC in addition to usual care for mental health disorders in epilepsy to assessment-enhanced usual care alone.

Patients aged 3–18 years within epilepsy services (and their accompanying parent/carer) will be approached. The process of determining eligibility will involve being asked some brief questions, completing the Strengths and Difficulties Questionnaire (SDQ) [25, 26] and, if above the threshold, providing informed consent before completing a full computerised psychiatric diagnostic assessment (the Development and Wellbeing Assessment: DAWBA [27]). Those meeting diagnostic criteria for a mental health disorder according to this assessment will be invited to take part in the trial. Participants will be individually randomised to usual care only or to usual care plus the MATCH-ADTC intervention. The primary outcome measure is the total difficulties score from the SDQ reported by the parent/carer at 6 months post-randomisation. The primary analysis will be conducted on an intention-to treat basis and blind to treatment assignment. All measures will be repeated 1 year post-randomisation to examine whether any improvements in mental health are maintained as a secondary outcome.

## Methods: participants, interventions and outcomes

### Study setting {9}

Recruitment will be from any epilepsy clinic setting, ranging from highly specialist to general hospitals, and from a range of populations served. All sites are in the UK. Therapists will be NHS staff working within epilepsy services and will include epilepsy nurse specialists, pre-qualification psychology assistants, neurologists, neuropsychologists and support workers. A full list of participating sites is available from http://www.isrctn.com/ISRCTN57823197.

### Eligibility criteria {10}

Participants will be considered eligible for enrolment in this trial if they fulfil all the inclusion criteria and none of the exclusion criteria as defined below.

#### Participant inclusion criteria

1. Attending NHS epilepsy clinics.
2. Aged 3–18 years.
3. Scoring above the pre-specified threshold on the SDQ for mental health symptoms which is a combination of raised total difficulty score (≥ 14) and raised impact score (≥ 2).
4. Meeting DSM-5 diagnostic criteria for a mental health disorder (e.g. depression, anxiety, disruptive behaviour or trauma) identified by the SDQ, the Development and Wellbeing Assessment (DAWBA) and clinical assessment.
5. Have a parent/carer who is also willing to take part in the study.

#### Participant exclusion criteria

1. Not speaking/understanding English sufficiently well to access the screening assessments.
2. Having an intellectual disability at a level meaning that they cannot access the measures or intervention.
3. Screening results that indicate a severe mental health disorder not considered suitable for the trial intervention due to the clinical need for immediate intervention, e.g. active suicidality and psychosis.
4. Actively receiving intensive psychological input focused on cognitive and/or behavioural strategies to intervene with emotional or behavioural difficulties at the time of the assessment or due to have such input during active phase of treatment.
5. Refusing to consent to the research team contacting their general practitioner (GP)/other relevant health professionals about their inclusion in the research.
6. Refusing to have the trial therapy sessions audio and/or video recorded.
7. Unable to complete the measures despite all reasonable efforts being made to assist.

### Who will take informed consent? {26a}

The order of screening procedures, consent and pre-randomisation assessments is as follows:

1. Families may be approached in clinic/hospital by a member of the research team and asked a few brief questions to determine their eligibility for participation and depending on their answers to these questions, they may be asked to complete the SDQ. Families may also be identified by clinicians who are familiar with them. In the latter situation, the clinical team will make contact with the family to ask them if they are interested in being contacted by the MICE trial team or wish to contact the MICE team directly. They will be asked the same questions to establish eligibility.
2. The parent (and child) must give verbal consent to complete the SDQ and agree to being contacted with the results. They will be offered the choice of completing the SDQ in clinic, online or alone in their own time or with support (via the telephone/online or in person).
3. Once the SDQ is completed, a member of the research team will inform the family of the results by telephone, letter, in person or secure email.
4. If the results of the SDQ indicate that the participant may be suitable for the trial, the parent/carer will be given the participant information sheet (PIS) and Informed Consent Form (ICF). The participant will be given an age appropriate PIS according to their developmental age and an ICF or Assent Form.
5. Written informed consent will be obtained from the parent/carer. Written informed consent or assent will be obtained from the participant. Participants aged 16 to 18 years must provide written informed consent if able to do so. If a participant is aged 16 to 18 years and lacks capacity to consent for themselves then their parent/carer should provide written informed consent on the participants’ behalf. Participants aged under 16 years should provide written informed assent if appropriate. The informed consent and assent process may take place by telephone but written informed consent/assent must subsequently be provided to the trial team (either by post, email or at a visit for trial records).

### Additional consent provisions for collection and use of participant data and biological specimens {26b}

The consent form includes consent for future contact regarding ancillary studies, including those related to data linkage with national datasets.

### Explanation for the choice of comparators {6b}

One potential advantage of MATCH-ADTC over existing protocols is that it can be used to treat the significant proportion (approximately 40%) of patients who have multiple mental health problems [18]. Other advantages are that the intervention is designed to be adapted for a diverse range of children, ages and problems and can be delivered in a range of social and healthcare services, including neurological services, by non-mental health specialists. In addition, it can be delivered over the telephone or via virtual online video platforms. The comparison against assessment-enhanced usual care ensures that we can determine the added value over existing service provisions.

### Intervention description {11a}

#### Assessment-enhanced usual care

**Fig. 1:**
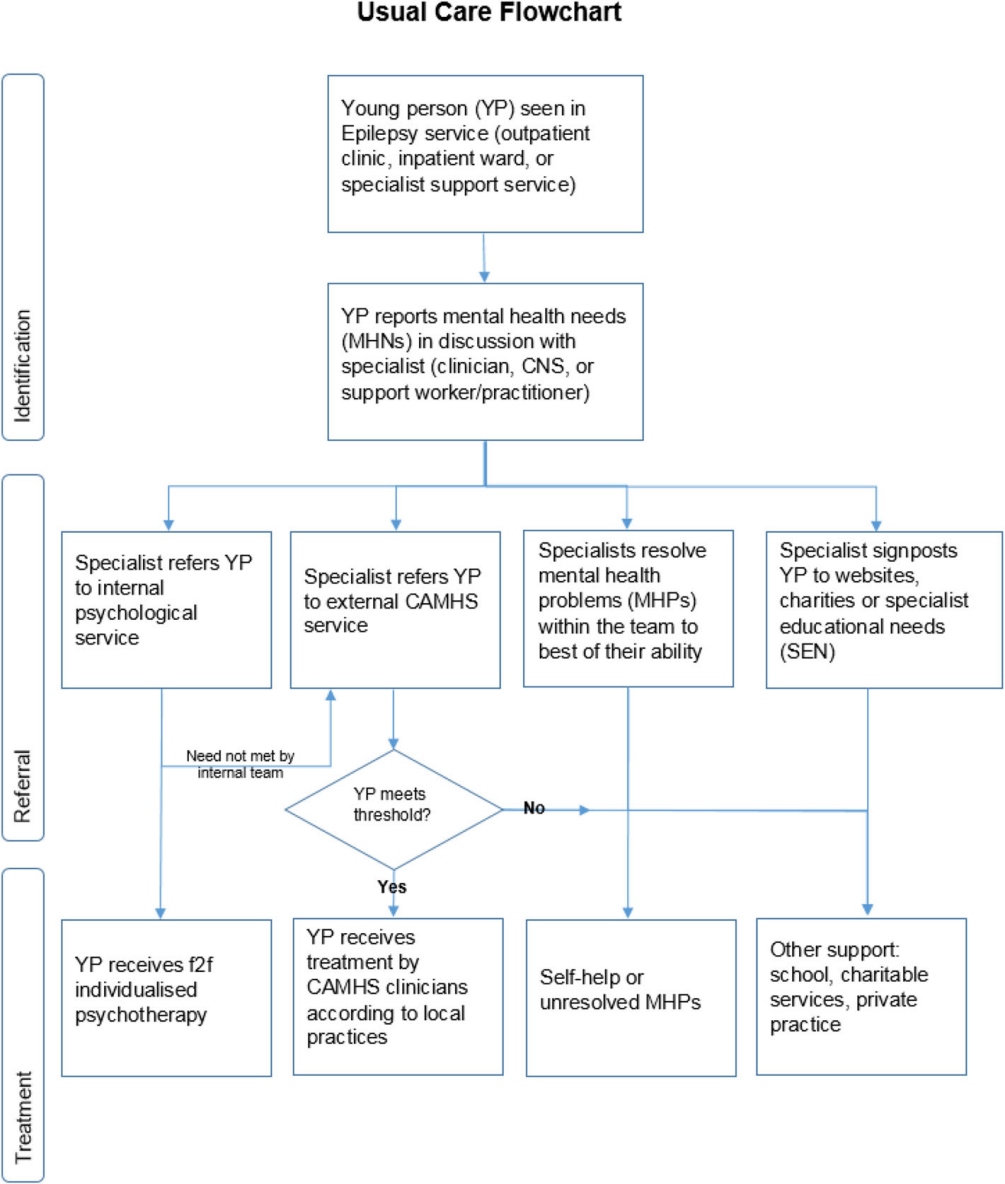
Usual care for mental health disorders in children and children and young people with epilepsy

Usual care for mental health disorders varies by site but typically includes referral to CAMHS or hospital-based paediatric psychology services (see Fig. 1). This intervention is considered as ‘assessment-enhanced’ usual care since those in this arm will have completed the DAWBA and the results of the DAWBA will be provided as information to the GP, referrer and other clinical team members involved as appropriate (e.g. neurologist or paediatrician).

#### MATCH-ADTC in addition to usual care

The epilepsy-specific version of MATCH-ADTC used in the trial is a personalised modular cognitive-behavioural intervention with epilepsy-relevant content integrated throughout and an additional epilepsy-specific focus area, including an epilepsy-specific compulsory module and three optional epilepsy-related modules delivered over the telephone/video conferencing for young people attending epilepsy clinics who meet diagnostic criteria for a DSM-5 mental health disorder, in addition to usual care.

MATCH-ADTC involves a weekly phone/online video call with the therapist who carries out the initial face-to-face assessment; parents complete measures at home and email them to the therapist before each session, and strategies are practiced at home. Face-to-face therapy sessions are permitted if clinically indicated or strongly preferred by the family. MATCH-ADTC comprises an average of 16 sessions but there is flexibility as many young people will have multiple mental health disorders whereas others may have one. MATCH-ADTC uses an algorithm to tailor the treatment to each young person’s individual needs and participants with co-occurring mental health disorders can have each of those addressed within the intervention. Those with intellectual disabilities may need to go at a slower pace than others and therefore may require more sessions. The minimum number is expected to be 10 and the maximum number of sessions is 22. The intervention must be completed within a 6-month window. The two booster sessions may be completed outside of this window. Some patients may receive fewer than 10 sessions providing that there is mutual agreement that their goals have been reached. All therapy sessions will be delivered within 6 months of randomisation although booster sessions can occur between 6 and 12 months post-randomisation. Clinical sessions including face-to-face sessions and telephone/online video calls will be audio and/or video recorded to ensure the sessions are delivered consistently. Key content of MATCH-ADTC for depression includes cognitive and problem-solving strategies and scheduling pleasurable activities; anxiety is addressed using exposure techniques; behavioural strategies, delivered through the parents, include one-on-one time, praise, effective instruction-giving, rewards and ignoring unwanted behaviour; trauma is addressed through developing a trauma narrative, exposure and safety planning. Session by session measurement of symptoms and progress towards self-identified goals is also included as part of the intervention.

Children and young people in the MATCH-ADTC arm will also access usual care for the mental health disorder if required as per Fig. 1.

### Patient public involvement (PPI)

There is a strong PPI element to the MICE trial, including a diverse and committed research advisory group (RAG) comprising parents, carers and young people with epilepsy (with an age range of 6–15 years). Meetings were held every 2 months to develop the treatment and quarterly during the training phase and will continue quarterly for the duration of the trial. Several members of the group were also involved in the advisory work for the programme development grant which preceded the MICE trial. All adult members of the RAG have completed modules 1 and 2 of NIHR online PPI training and several of the group have co-authored a paper on patient experiences which has been submitted for publication. The PPI lead was part of the interview panel which recruited two research assistants to the project and has co-presented at an event with the PI about the impact of high-quality PPI. The Steering Group also has two independent PPI representatives with lived experience. The goals of the PPI group are to ensure that the trial meets the needs of the population it is intended to serve.

### Criteria for discontinuing or modifying allocated interventions {11b} –

In consenting to the trial, participants are consenting to trial treatment, trial follow-up and data collection. However, an individual participant may stop treatment early or be stopped early for any of the following reasons:

- Unacceptable serious adverse event
- Inter-current illness that prevents continuing the trial intervention
- Any change in the participant’s condition that in the clinician’s opinion justifies the discontinuation of the trial intervention
- Withdrawal of consent for the trial intervention by the participant

As participation in the trial is entirely voluntary, the participant may choose to discontinue trial treatment at any time without penalty or loss of benefits to which they would otherwise be entitled.

### Training and supervision of therapists

Professionals from a range of backgrounds (including consultant paediatricians, paediatric nurses, assistant psychologists and epilepsy nurse specialists) were trained over a 6-month period to deliver the mental health intervention. Training included a 5-day intensive training led by experts in the field of epilepsy and mental health, including members of the MATCH-ADTC team, epilepsy experts and mental health professionals with extensive experience in working with children, young people and families. Therapists received a half-day booster training at the end of the 6-month period with topics tailored to their individual training needs. All therapists saw at least one family for telephone-delivered mental health treatment during the 6-month training period during which therapists were offered weekly telephone clinical supervision with a qualified clinical psychologist. Supervision-of-supervision was provided on a monthly basis by a member of the MATCH-ADTC team.

### Strategies to improve adherence to interventions {11c}

Therapist competence will be assessed using the same methods as successfully applied to earlier studies of MATCH-ADTC. Fidelity and competence will be established by expert review of 10% of the sessions selected at random by a BABCP-accredited therapist and rated using the Cognitive Therapy Rating Scale Revised (CTS-R) and in accordance with the principles of MATCH-ADTC [28, 29] throughout the duration of the trial. In addition, 10% of these will be selected at random and double-rated by an external expert in the USA who has been accredited by the developers of the intervention as a supervisor and trainer.

### Relevant concomitant care permitted or prohibited during the trial {11d}

Participants who are currently actively receiving intensive psychological input focused on cognitive and/or behavioural strategies to intervene with emotional or behavioural difficulties, or due to have such input within 6 months of randomisation, will not be included in the trial.

### Provisions for post-trial care {30}

There are no plans for ancillary or post-trial care. Any significant risk issues will be communicated to the families’ GP and any other clinician involved in their care. Referrals or requests for referrals to other services may be made as appropriate.

Cover for negligent harm will be provided by the Great Ormond Street Hospital for Children (GOSH) NHS Foundation Trust through the Clinical Negligent Scheme for Trusts (CNST) and University College London (UCL).

Participants may also be able to claim compensation for injury caused by participation in this clinical trial without the need to prove negligence on the part of the sponsor or another party. Participants who sustain injury and wish to make a claim for compensation should do so in writing in the first instance to the Chief Investigator (CI), who will pass the claim to the sponsor’s office.

Hospitals selected to participate in this clinical trial shall provide clinical negligence insurance cover for harm caused by their employees and a copy of the relevant insurance policy or summary shall be provided to UCL, upon request.

### Outcomes {12}

#### Primary outcome

The primary outcome measure is the total difficulties score from the Strengths and Difficulties Questionnaire (SDQ) [25, 26] reported by the parent/carer at 6 months post-randomisation. The SDQ is a brief behavioural screening questionnaire for children and young people aged 2 years and above. The 25 items are divided between 5 scales (emotional, conduct problems, hyperactivity/inattention, peer relationship problems, prosocial behaviour), with the first four added to provide a total difficulties score. The SDQ has good internal consistency (mean Cronbach *α* .73), cross-informant correlation (mean 0.34), and retest stability after 4 to 6 months (mean 0.62) [30]. SDQ scores above the 90th percentile predict a substantially raised probability of independently diagnosed psychiatric disorders (mean odds ratio 15.7 for parent scales) [30]. It has been validated across the age range of children and young people seen within neurology clinics and used in those with autism spectrum disorder [31] and intellectual disabilities [32] known to be highly prevalent in this group [33]. Further, the SDQ has an ‘impact scale’, which assesses the impact that symptoms have on everyday life in a range of domains (home, school, leisure). The parent-reported measure was chosen for data completeness, as the self-report version is unsuitable for young people under the age of 11 years.

#### Secondary outcomes

- Revised Child Anxiety and Depression Scale (RCADS) [34]. This 47-item questionnaire is one of the main outcome measures used in the Children and Young People’s Improving Access to Psychological Therapies (CYP-IAPT) Programme. It is included to allow comparison with this national initiative and has parent and child versions, the subscales are reliable (Cronbach *α* .78–.88) and the measure has discriminant, convergent and factorial validity in a clinical sample [35]. For the trial, the parent version will be used for data completeness.
- Patient Health Questionnaire (PHQ-9) [36]. This is a 9-item measure of depression in adults completed by the parent/carer about their own mental health.
- Generalised Anxiety Disorder Assessment (GAD-7) [37]. This is a 7-item measure of generalised anxiety disorder, completed by the parent/carer about their own mental health.
- Hague Seizure Severity Scale (HASS) [38]—this is a parent-report questionnaire which rates carers’ subjective experiences of the severity of their child’s seizures. Items are statements, which are rated on a 4-point scale from most to least severe (e.g. ‘always’, ‘usually’, ‘sometimes’ or ‘never’). Items describe the length, type and outcomes of a seizure—for example ‘how long do the jerks or cramps last during an attack?’
- Paediatric Quality of Life Epilepsy Module (PedsQL) [39]. The PedsQL measures the impact of epilepsy on quality of life.
- Number of serious adverse events. Serious adverse events are those that result in death, are life threatening, require hospitalisation or prolong existing hospitalisation, result in persistent or significant disability or incapacity, or result in a congenital anomaly or birth defect or another important medical condition.

#### Health economic measures

The following measures will be reported by the parents:

CA-SUS—Service use measured using the Child and Adolescent Service Use Schedule, developed and applied in a range of populations of young people with mental health problems [40, 41].

CHU-9D—Child Health Utility 9-dimensions [42]. This is a paediatric health-related quality of life measure for use in economic evaluation.

EQ-5D-5L—EuroQol 5-dimensions, 5-level version [43–46]. The 5-level EQ-5D comprises 5 dimensions (mobility, self-care, usual activities, pain/discomfort and anxiety/depression) and will be used to assess the health-related quality of life of the main parent/carer.

Additionally, indirect (non-face-to-face) time spent on different activities in a typical week will be estimated by therapists using a questionnaire to support the costing of the intervention.

### Participant timeline {13}

See Table 1.

**Table 1:**
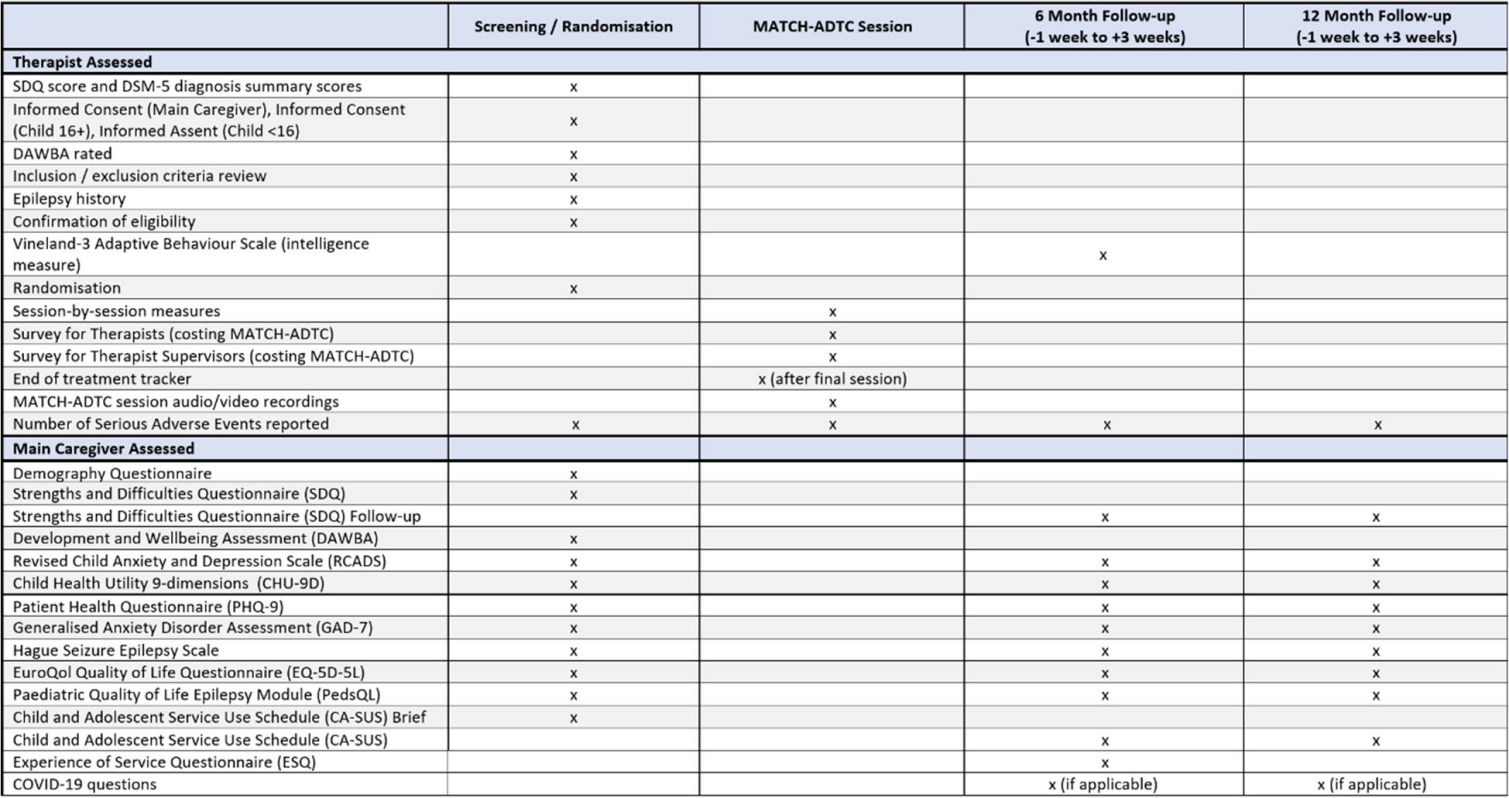
Participant timeline for assessment and measure completion

### Sample size {14}

The sample size is 334 children and young people with epilepsy, and their parents /carers.

Previous effect size (ES; standardised magnitude of the effect) estimates for MATCH-ADTC using measures comparable to the SDQ range from 0.51 to 0.65 [14, 15]. Previous published research has estimated the ES for usual care in UK CAMHS to be 0.16–0.2 [47]. We have therefore conservatively based our calculation on an ES = 0.3 which is modest for a psychological intervention study. Our small pilot study comparing the guided self-help psychological intervention against a waitlist control found a large effect size.

A total sample size of 334 children has been chosen as this could detect an effect size of 0.3 for the SDQ, at the 5% significance level with 80% power, assuming an average of 14 children per therapist, an intraclass correlation coefficient (ICC) of 0.01 for therapist effects, a correlation of 0.5 between baseline and follow-up SDQ, and a loss to follow-up rate of 10%. We will take steps to minimise the amount of missing data. A generalised mixed model will be used to analyse the primary outcome adjusting for baseline SDQ and minimisation variables.

### Recruitment {15}

Clinicians can directly refer potential participants to the study. As research has demonstrated that mental health disorders are frequently not detected in children with epilepsy, the study also includes a screening component in which all patients attending epilepsy clinics and meeting inclusion criteria will be invited to complete screening measures and proceed further with the study according to the results of this screening. Please see section on informed consent for further details.

## Assignment of interventions: allocation

### Sequence generation {16a}

Patients will be randomised in a 1:1 ratio to MATCH-ADTC plus usual care or to assessment-enhanced usual care alone, using an independent web-based online system (https://www.sealedenvelope.com). Randomisation will use a minimisation algorithm incorporating a random element, stratifying using the following factors:

- Primary mental health disorder—anxiety/depression/disruptive behaviour/trauma
- Presence of autistic spectrum disorder or autism—yes/no
- Age—< 11/11+
- Presence of intellectual disability—yes/no

Primary mental health disorder and the presence of autism spectrum disorder will be decided according to the results of the DAWBA. Presence of intellectual disability will be self-reported by parents at baseline; this self-report will be considered in the context of scores on the Vineland Adaptive Behaviour Scales (VABS) [48] at 6 months post-randomisation. These factors were chosen due to the high associations between epilepsy and autism spectrum disorder and intellectual disability [49] and the desire to have equal proportions of primary mental health disorder and ages in the two treatment allocations. Each patient will be randomised using their unique participant identification number that was allocated sequentially at screening. Eligibility and consent will be verified before each patient is randomised.

### Concealment mechanism {16b}

The allocation will be concealed prior to assignment to prevent allocation bias.

### Implementation {16c}

Eligibility and consent will be confirmed before each participant is randomised.

## Assignment of interventions: blinding

### Who will be blinded {17a}

Trial participants, their parent/carer, therapists and the MICE study team will be unblinded to treatment allocation.

Participant outcome assessments at 6 months (the primary endpoint) and 12 months will be carried out by an independent researcher blind to treatment allocation.

The final analysis will be performed by a blinded statistician. An unblinded statistician at the Comprehensive Clinical Trials Unit at UCL (CCTU) will prepare any sections of reports for the data monitoring and ethics committee (DMEC) in which data is presented by treatment arm.

Health economic analysis cannot be undertaken blind because the analysis requires the intervention cost to be applied to those young people in the intervention group. However, health economic analysis results will be presented to and interpreted by the Programme Management Group blind to allocation (i.e. results will be presented as group A versus group B).

### Procedure for unblinding if needed {17b}

This is not needed as trial participants, their parent/carer, therapists and the MICE study team will be unblinded to treatment allocation.

## Data collection and management

### Plans for assessment and collection of outcomes {18a}

Each participant will be given a unique trial participant identification number (PIN). Data will be collected at the time-points indicated in the participant timeline.

All data will be entered on the database either by parents/carers, by members of staff delegated to work on the trial within each research site or members of the MICE trial team based at GOSH ICH. The preferred method of data collection is for data to be entered directly into the Sealed Envelope database (www.sealedenvelope.com). It will be possible for parents/carers to self-complete some forms directly on the database. Investigators will be able to use the database to send an email or text message link to invite parents/carers to self-complete relevant forms. If self-completion online is not possible, site staff can ask the parents/carers the questions by telephone and enter the data directly. If this is not possible, then parents/carers are able to complete paper forms/questionnaires which can then be entered on to the database by site staff.

Paper worksheets will be provided as aides for data collection; however, the completion of these is optional. Completion of the worksheets may be helpful if a computer is not readily available to ensure all required data is collected. If data is collected on paper worksheets, e.g. from clinical studies officers, data entry will be performed as soon as possible by a member of the MICE study team. We will record whether data were collected directly from the participant or whether it was entered by a member of the MICE study team.

Data collection, data entry and queries raised by a member of the MICE trial team will be conducted in line with the CCTU and trial specific Data Management Plan.

Clinical trial team members will receive trial protocol training. All data will be handled in accordance with the General Data Protection Regulation and UK Data Protection Act 2018.

### Data management {19}

Data will be entered using the participant’s PIN onto a central database hosted by the third-party organisation Sealed Envelope. The database will be password protected and only accessible to parents/carers and participants (where appropriate), delegated members of site staff and the MICE trial team, and external regulators if requested.

The Sealed Envelope database is hosted on servers in the UK provided by Rackspace UK; these servers are backed up by Amazon Web Services Ireland (AWS), and both Rackspace UK and AWS have been certified to ISO 27001 and ISAE 3402. Access to the database is controlled by password-protected accounts, and an individual’s access to the database is restricted by their role. The underlying database infrastructure is encrypted and access is strictly limited to Sealed Envelope staff.

The database and metadata have been developed by the clinical trial manager in conjunction with CCTU. The database software provides a number of features to help maintain data quality, including maintaining an audit trail, allowing custom validations on all data, allowing users to raise data query requests and reports to identify validation failure/missing data.

The identification, screening and enrolment logs, linking participant identifiable data to the pseudoanonymised PIN, will be held locally by the trial site. This will either be held in written form in a locked filing cabinet or electronically in password-protected form on hospital computers. After completion of the trial, the identification, screening and enrolment logs will be stored securely by the sites for 10 years unless otherwise advised by CCTU.

### Plans to promote participant retention and complete follow-up {18b}

The importance of attending scheduled therapy sessions and follow-up visits until trial completion will be explained to all participants at the start of the trial to ensure that only those able to commit to the protocol are recruited. Participants who discontinue protocol treatment should remain in the trial for the purpose of follow-up and data analysis. They will be asked whether they continue to consent to being contacted for follow-up assessments. Data will be analysed on an intention-to-treat (ITT) basis. Therefore, withdrawal from the study will not result in exclusion of the data for that participant from analysis. Participants who are randomised but discontinue trial treatment or withdraw from the trial will not be replaced.

### Confidentiality {27}

Patient identifiable data, including initials, and date of birth will be required for the registration process. The study staff will ensure that the participants’ anonymity is maintained, except where information is disclosed by a patient or parent/carer that suggests a risk of harm to the patient, parent/carer or others. Each participant will be assigned a unique 5-digit trial PIN by site staff. The database will record the PIN, the patient’s initials and date of birth, but not the patient’s name. The only link between the PIN and the patient’s name will be on the screening log kept at site and accessed only by the patient’s direct clinical care team. Data will be recorded on the custom-designed Sealed Envelope database under this identification number. The database will be password protected and only accessible to members of the MICE trial team at CCTU, trained and authorised site staff, and external regulators if requested.

All documents will be stored securely and only accessible by study staff and authorised personnel. The study will comply with the Data Protection Act, which requires data to be anonymised as soon as it is practical to do so.

Data will be stored in a secure manner and in accordance with the Data Protection Act 2018.

The research team will have access to participants’ medical records (including their hospital records). Access to participants’ medical history and to files to write summary notes of progress will be necessary. Access to medical records by those outside of the direct healthcare team will only be gained after the participant/parent/carer has given their consent for this. Participants’ educational records may also be accessed by the research team. Consent will be taken for potentially linking data collected to Health Episode Statistics and the National Pupil Database.

Postal and email addresses will be required to write to participants (for example to send them progress reports or to send them electronic copies of measures). Phone numbers and/or usernames/addresses will be required for telephone/video conferencing consultations as part of the intervention, possibly for interviews and assessments and to provide support in completing measures and to send text messages. The research team will only have access to these details if the participant has consented to this, and such data will be held at Great Ormond Street Hospital. All phone/video conference calls and face-to-face meetings, including assessments, interviews and clinical sessions, will be audio/video recorded. Only therapists will be shown on video recordings.

Audio recording of some participants’ assessment interviews will be transcribed verbatim and used for the purposes of qualitative analysis. Quotes from these interviews may be used to illustrate presenting themes, but any data that would make the participant identifiable would be removed. Telephone calls will also be audio recorded, for use in supervision and to ensure fidelity to the protocol. Video recordings of clinical sessions may also be made. Only therapists will be shown on video recordings. Personal data will be contained within a project master file (paper copy), which will be kept in a locked cabinet accessible only to the research team at GOSH. Personal data contained on research databases (for example participant contact addresses) will be kept on a secure, restricted-access drive, only accessible to the research team with GOSH contracts.

All other electronic information pertaining to the trial will be kept in encrypted, password-protected files stored on the GOSH internal network (including GOSH laptops). In the event of a sponsor-led audit or inspection, the authorised research and development (R&D) individual will also require access to the participant’s medical and educational records in the course of their duties.

## Statistical methods

### Statistical methods for primary and secondary outcomes {20a}

Patient characteristics at the time of randomisation will be summarised using mean and SD for continuous variables which are approximately normally distributed, median and interquartile range (IQR) for variables which are not normally distributed, or by frequencies and percentages for categorical variables. All statistical hypothesis tests will use a two-sided *p* value of 0.05 unless otherwise specified. All confidence intervals (CIs) presented will be 95% and two-sided. All statistical analysis will be performed using Stata (StataCorp, College Station, TX, USA) or an alternative package.

#### Primary outcome

The primary outcome is the difference between the intervention group and the control group in the total difficulties score of the parent-reported SDQ at 6 months post-randomisation.

The total difficulties score of the SDQ has a range of 0–40. Mixed effect linear regression will be used to determine if there is any difference in the total difficulties score due to intervention. The model will include fixed effects for intervention group, baseline SDQ total difficulties score and the stratification factors; age (< 11 versus 11+), primary mental health disorder (anxiety, depression, disruptive behaviour or trauma), presence of autistic spectrum disorder (yes/no) and presence of intellectual disability (yes/no). A random factor, *Therapist*, will be included to take account of therapist effects. Results will be presented as adjusted treatment effect and the associated 95% CI. If the intervention is effective, we would expect to see a reduction in the total difficulties score (higher total difficulties score indicates a higher risk of a diagnosis of mental illness).

The primary analysis will be conducted following the intention-to-treat principle in accordance with the randomised intervention. All efforts will be made to ensure that the primary outcome data is collected for all patients, whether or not they complete their randomised treatment. Missing baseline data are not anticipated since baseline data are completed prior to randomisation and must be recorded to allocate treatment. All patients with reported outcome data will be included in the analysis. The primary analysis is likelihood based and therefore robust to the assumption of missing-at-random, but should substantial missing data be encountered, reasons for missingness will be explored and a sensitivity analysis will be undertaken to investigate the validity of the missing at random assumption.

#### Secondary outcomes

Separate analyses will be performed for outcomes at 6 months and at 12 months.

The timing of the primary effectiveness outcome at 6 months is because we expect to see the maximum benefit of treatment at this time point. However, we will be following up the children and the difference at 12 months is a secondary outcome.

Models for all secondary efficacy outcomes will be adjusted for the stratification factors.

Binary secondary outcomes will be analysed using mixed effects logistic regression, ordinal secondary outcomes with mixed effects ordinal logistic regression, and continuous secondary outcomes with mixed effects linear regression.

Fisher’s exact test will be used to compare the proportion of children with any serious adverse events in the two randomised groups.

### Economic analysis

A detailed health economic analysis plan, including a full specification of the analysis principles and details, will be written prior to the first substantive analysis and approved in advance by the programme steering committee (PSC).

Costs and cost-effectiveness will be compared between groups at the final 12-month follow-up to capture the economic impact over the longest period available. A secondary analysis at the 6-month follow-up will be carried out for consistency with the primary clinical analysis; however, to avoid unblinding the analyst earlier than necessary, this will be carried out after the primary economic analysis has been completed.

The economic perspective will be that of the NHS and personal social services sectors including those provided within the education sector. Health and social care service use will be collected using the CA-SUS at baseline (covering the previous 3 months) and at the 6- and 12-month follow-up points (covering the period since last interview) using the CA-SUS. Service use will be valued using nationally applicable unit costs (NHS Reference Costs and Personal Social Service Research Unit (PSSRU) Costs of Health and Social Care). The intervention will be directly costed taking a bottom-up (micro-costing) approach and using data on direct contacts recorded in clinical records. Indirect (non-face-to-face) time will be estimated using a questionnaire completed by therapists on time spent on different activities in a typical week. Intervention costs will be estimated using information on therapist salaries and working conditions, including relevant overhead costs (capital, managerial, administrative, etc.).

As a result of current limitations in the measurement of health-related quality of life in young children, capable of generating quality-adjusted life years (QALYs) for application in a cost-utility analysis, our primary economic analysis will be a cost-effectiveness analysis using the primary clinical outcome measure (parent-reported SDQ total difficulties score).

A more exploratory secondary economic analysis will consider cost-utility analysis using the CHU-9D to generate QALYs. Parents of all participants will be asked to proxy-complete the CHU-9D on behalf of all young people.

Additionally, self-report health-related quality of life data will be collected for the main parent/carer using the EQ-5D-5L to explore the impact of the intervention on both young people and their carers.

Cost-effectiveness will be assessed using the net benefit approach and following standard approaches [50]. A joint distribution of incremental mean costs and effects for the two groups will be generated using nonparametric bootstrapping to explore the probability that each of the treatments is the optimal choice, subject to a range of possible maximum values (ceiling ratio) that a decision-maker might be willing to pay for an additional unit of outcome gained. Cost-effectiveness acceptability curves will be presented by plotting these probabilities for a range of possible values of the ceiling ratio [51]. These curves are a recommended decision-making approach to dealing with the uncertainty that exists around the estimates of expected costs and expected effects associated with the interventions under investigation and uncertainty regarding the maximum cost-effectiveness ratio that a decision-maker would consider acceptable.

All economic analyses will be adjusted for co-variates in line with the clinical analyses described above, including the stratification variables (age (< 11 versus 11+), primary mental health disorder (anxiety, depression, disruptive behaviour or trauma), presence of autistic spectrum disorder (yes/no), presence of intellectual disability (yes/no) and for the baseline values of the variables of interest (e.g. baseline cost, CHU-9D score, as appropriate).

### Interim analyses {21b}

No interim analyses are planned by the trial team (TT). Regular reports will be provided to the DMEC members.

### Methods for additional analyses (e.g. subgroup analyses) {20b}

Planned subgroup analyses will be conducted to investigate whether the effect of treatment varies between patients with regard to each of the stratification factors. This will be explored by adding an interaction between each of the factors and treatment to the primary outcome analysis model separately.

Potential mediators and moderators will also be explored. Further details on potential factors and the appropriate analysis methods will be considered in the SAP.

### Methods in analysis to handle protocol non-adherence and any statistical methods to handle missing data {20c}

The main analysis will be conducted following the intention-to-treat principle in accordance with the randomised intervention. All patients with outcome data will be included in the analysis. The primary analysis is likelihood based and therefore robust to the assumption of missing at random, but should substantial missing data be encountered, reasons for this will be explored and a sensitivity analysis will be undertaken to investigate the validity of this missing at random assumption. If the missing at random assumption appears reasonable and the loss to follow-up rate is as anticipated, then we would not use multiple imputation. However, this will be considered in detail in the statistical analysis plan (SAP).

### Plans to give access to the full protocol, participant level-data and statistical code {31c}

The Chief Investigators, Clinical Project Manager, Trial Manager, Statistician, Health Economist and Trial Management Team will have full access to the trial data.

## Oversight and monitoring

### Composition of the coordinating centre and trial steering committee {5d} –

The composition and terms of reference of committees are available on request from the Chief Investigators.

#### Trial Team (TT)

The MICE TT will be responsible for the day to day management of the trial, including operational issues and budget management.

#### Coordinating Centre

The CCTU at UCL act as coordinating centre for the trial, providing specialism in trial design, conduct and statistics. CCTU will also be responsible for monitoring activities undertaken on behalf of the sponsor.

#### Programme management group

A programme management group (PMG) assists with developing the design, co-ordination and strategic management of the trial.

#### Programme steering committee

The PSC is the independent group responsible for oversight of the trial in order to safeguard the interests of trial participants. The PSC provides advice to the CI, CCTU, the funder and sponsor on all aspects of the trial through its independent Chair.

### Composition of the data monitoring committee, its role and reporting structure {21a}

The independent data monitoring and ethics committee (DMEC) is the only oversight body that has access to accumulating comparative data. The DMEC is responsible for safeguarding the interests of trial participants, monitoring the accumulating data and making recommendations to the PSC on whether the trial should continue as planned. The membership, frequency of meetings, activity (including review of trial conduct and data) and authority will be covered in the DMEC terms of reference. The DMEC will consider data in accordance with the statistical analysis plan and will advise the PSC through its Chair. The composition and terms of reference of the DMEC are available on request from the Chief Investigators.

### Adverse event reporting and harms {22}

For the purposes of the MICE trial, only serious adverse events will be reported. All serious adverse events (SAEs) occurring during the trial observed by the investigator or reported by the participant, whether or not attributed to the trial treatment will be recorded on the database. Low-grade adverse events that do not meet the seriousness criteria will not be reported, for example a grade 1 or 2 epileptic seizure that does not result in hospitalisation.

Investigators should notify CCTU immediately and within 24 h at the latest of any SAEs occurring from the time of randomisation until 30 days after the participant completes trial treatment. From this point forward, the site will notify the CCTU of SAEs that are considered related to the trial intervention if they become aware of them until end of follow-up.

Participants must be followed up until clinical recovery is complete, or until the event has stabilised. Follow-up should continue after completion of trial treatment and/or trial follow-up if necessary. Follow-up SAE forms (clearly marked as follow-up) should be completed and emailed to CCTU as further information becomes available. Additional information and/or copies of test results, etc., may be provided separately.

Medically qualified staff appointed by the sponsor will review all SAE reports received. The reviewer will complete the assessment of expectedness.

SAEs that are considered to be related to the trial (resulted from administration of any of the research procedures) and unexpected must be reported to the REC and DMEC. If the related and unexpected event is fatal or life threatening, it must be reported within 7 days of becoming aware of the event; other related and unexpected events must be reported within 15 days.

A list of expected occurrences as a result of the trial treatment is as follows:

- Significant deterioration in behaviour operationalised as consistent decline for three consecutive weeks, over four measurement time points, of at least 5 points for relevant behaviour based goals (e.g. as a result of an ‘extinction burst’)
- Significant deterioration in anxiety operationalised as consistent decline for three consecutive weeks, over four measurement time points, of at least 5 points for relevant anxiety-based goals (e.g. as a result of exposure to feared situations)
- Significant deterioration in mood operationalised as consistent decline for three consecutive weeks, over four measurement time points, of at least 5 points for relevant mood-based goals (e.g. as a result of cognitive challenging)

Where the SAE falls outside of this list, it must be deemed to be unexpected by the clinical reviewer.

CCTU will keep investigators informed of any safety issues that arise during the course of the trial.

### Frequency and plans for auditing trial conduct {23}

CCTU staff will review data entered on the database for errors and missing key data points. The trial database will also be programmed to generate reports on errors and error rates. Data quality issues will regularly be communicated to the TT. Essential trial issues, events and outputs, including defined key data points, will be detailed in the MICE trial Data Management Plan.

The frequency, type and intensity of routine and triggered on-site monitoring conducted by CCTU on behalf of the sponsor will be detailed in the MICE Quality Management and Monitoring Plan (QMMP). The QMMP will also detail the procedures for review and sign-off of monitoring reports.

### Plans for communicating important protocol amendments to relevant parties (e.g. trial participants, ethical committees) {25}

The CCTU ensure that the current trial protocol, patient information sheet, consent form, GP letter and submitted supporting documents have been approved by the REC and site R&D departments prior to use in patient recruitment. The protocol and all agreed substantial protocol amendments will be documented and submitted for ethical approval prior to implementation. Where changes are planned to patient-facing documentation, PPI groups will be consulted in advance for input.

## Dissemination plans {31a}

The dissemination plans follow best-practice recommendations. The plan is to engage with policy-makers, patients, carers, the public and media contacts to disseminate the findings across the public and private sectors and charities. We plan to publish the findings in peer-reviewed journals, and at national and international conferences. We will work with professional colleges, funders, the Epilepsy 12 national audit group and our PPI group to disseminate the findings widely to all stakeholders via traditional and social media.

## Response to COVID-19

The COVID-19 pandemic may have a significant impact on the mental health of participants. We therefore plan to ask a series of questions at 6 and 12 months post-randomisation to understand how participants and their families have been affected in order to help provide context to the findings of the study.

## Discussion

This protocol sets out the rationale and methods for evaluating the impact of adding a modular psychological intervention to usual care for young people with mental health disorders in the context of epilepsy. The psychological intervention will be delivered by non-mental health specialists from within epilepsy services in order to facilitate the closer integration of physical and mental health care. Given the high rates of mental health disorders coupled with high rates of unmet need in this group, positive results could result in suggestions for changes in service organisation and delivery in the UK and internationally. The model could also be used for the treatment of mental health disorders in children with other long-term conditions.

## Trial status

This manuscript presents the current version (4.0 of 27th July 2020) of the MICE Protocol. Recruitment commenced in May 2019. It was originally expected to be completed by May 2021 but due to delays resulting from the COVID-19 pandemic, is now anticipated to be completed by May 2022.

## Data Availability

Requests for access to trial data will be considered and approved in writing where appropriate, after formal application to the PSC. Considerations for approving access are documented in the PSC Terms of Reference (TOR).

## Abbreviations

AWS: Amazon Web Services
CA-SUS: Child and Adolescent Service Use Schedule
CAMHS: Child and Adolescent Mental Health Services
CBT: Cognitive behaviour therapy
CCTU: Comprehensive Clinical Trials Unit at UCL
CHU-9D: Child Health Utility 9-dimensions
CI: Chief Investigator
CIs: Confidence intervals
CNST: Clinical Negligent Scheme for Trusts
CTS-R: Cognitive Therapy Rating Scale Revised
CYP-IAPT: Children and Young People’s Improving Access to Psychological Therapies
DAWBA: Development and Well-being Assessment
DMEC: Data monitoring and ethics committee
DoH: Department of Health
DSM-5: Diagnostic and Statistical Manual of Mental Disorders
EQ-5D-5L: Five-dimension, five-level version of the EuroQol measure of health-related quality of life
ES: Effect size
GAD-7: Generalised Anxiety Disorder Assessment
GOSH: Great Ormond Street Hospital
GP: General Practitioner
HASS: Hague Seizure Severity Scale
ICC: Intraclass correlation coefficient
ICF: Informed consent form
ICH: Institute of Child Health
IQR: Interquartile range
ITT: Intention to treat
MATCH-ADTC: Modular Approach to Therapy for Children with Anxiety, Depression, Trauma or Conduct Problems
MICE: Mental Health Intervention for Children with Epilepsy
NHS: National Health Service
PASCET: Primary and Secondary Control Enhancement Training
PedsQL: Paediatric Quality of Life Epilepsy Module
PHQ-9: Patient Health Questionnaire
PIN: Participant identification number
PIS: Participant information sheet
PMG: Programme management group
PPI: Patient and public involvement
PSC: Programme steering committee
PSSRU: Personal Social Service Research Unit
QALY: Quality-adjusted life year
QMMP: Quality Management and Monitoring Plan
R&D: Research and development
RAG: Research advisory group
RCT: Randomised controlled trial
RCADS: Revised Child Anxiety and Depression Scale
REC: Research ethics committee
SAE: Serious adverse event
SAP: Statistical analysis plan
SDQ: Strengths and Difficulties Questionnaire
TT: Trial Team
ToR: Terms of Reference
UCL: University College London
VABS: Vineland Adaptive Behaviour Scales
